# A case of unresectable combined hepatocellular and cholangiocarcinoma treated with atezolizumab plus bevacizumab

**DOI:** 10.1002/ccr3.6129

**Published:** 2022-07-25

**Authors:** Naoto Saito, Takeshi Hatanaka, Sachi Nakano, Yoichi Hazama, Sachiko Yoshida, Yoko Hachisu, Yoshiki Tanaka, Teruo Yoshinaga, Kenji Kashiwabara, Norio Kubo, Yasuo Hosouchi, Hiroki Tojima, Satoru Kakizaki, Toshio Uraoka

**Affiliations:** ^1^ Department of Gastroenterology Gunma Saiseikai Maebashi Hospital Maebashi Japan; ^2^ Department of Pathology Gunma Saiseikai Maebashi Hospital Maebashi Japan; ^3^ Department of Surgery Gunma Saiseikai Maebashi Hospital Maebashi Japan; ^4^ Department of Gastroenterology and Hepatology Gunma University Graduate School of Medicine Maebashi Japan; ^5^ Department of Clinical Research National Hospital Organization Takasaki General Medical Center Takasaki Japan

**Keywords:** anti‐programmed death ligand‐1, hepatic resection, immune checkpoint inhibitor, liver cancer, vascular endothelial growth factor

## Abstract

An 81‐year‐old man initially underwent right hepatic lobectomy for liver cancer and was pathologically diagnosed with combined hepatocellular and cholangiocarcinoma (CHC). At 13 months after resection, multiple lymph node metastases were observed. We started atezolizumab plus bevacizumab (Atez/Bev), achieving a 7.5‐month progression‐free survival. Atez/Bev might exhibit efficacy for CHC patients.

## Graphical abstract

Although no standard systemic chemotherapy for combined hepatocellular and cholangiocarcinoma patients has yet been established, atezolizumab plus bevacizumab, which is a combination therapy of anti‐programmed death ligand‐1 and anti‐vascular endothelial growth factor, might exhibit good therapeutic outcome.

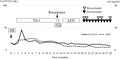

## 1 INTRODUCTION

Primary liver cancer is the sixth‐most commonly diagnosed cancer and the third leading cause of cancer death worldwide, with an estimated 906,000 new cases and 830,000 deaths annually.^1^ Combined hepatocellular and cholangiocarcinoma (CHC) is a rare form of primary liver cancer that shows the histopathological features of both hepatocellular carcinoma (HCC) and cholangiocarcinoma within the same tumor.^2^, ^3^ Surgical treatment may be preferred for patients with localized disease.^4^, ^5^ However, the disease commonly recurs during the post‐operative observation, often with unresectable regional or distant or metastatic disease.^6^, ^7^, ^8^ In addition, the number of patients receiving surgical treatment is limited, according to a large population‐based study.^9^

No standard systemic chemotherapy for unresectable patients has yet been established, as evidence has been limited due to the rarity and the heterogeneity of the disease. Accordingly, physicians offer a biliary tract cancer‐based regimen or an HCC‐based regimen for advanced CHC patients. There have been some studies exploring the efficacy and safety of certain regimens, including gemcitabine plus cisplatin, fluorouracil plus cisplatin, and sorafenib,^10^, ^11^, ^12^ but these studies were reported in a retrospective manner.

Recently, atezolizumab and bevacizumab (Atez/Bev), which is a combination therapy of anti‐programmed death ligand‐1 (PD‐L1) and anti‐vascular endothelial growth factor (VEGF), was introduced as the first immune‐combined therapy for patients with HCC and showed advantages over sorafenib in terms of the overall and progression‐free survival.^13^ However, the efficacy and safety of Atez/Bev for patients with CHC remains uncertain.

We herein report a case of unresectable CHC successfully treated with Atez/Bev.

## 2 CASE REPORT

**FIGURE 1:**
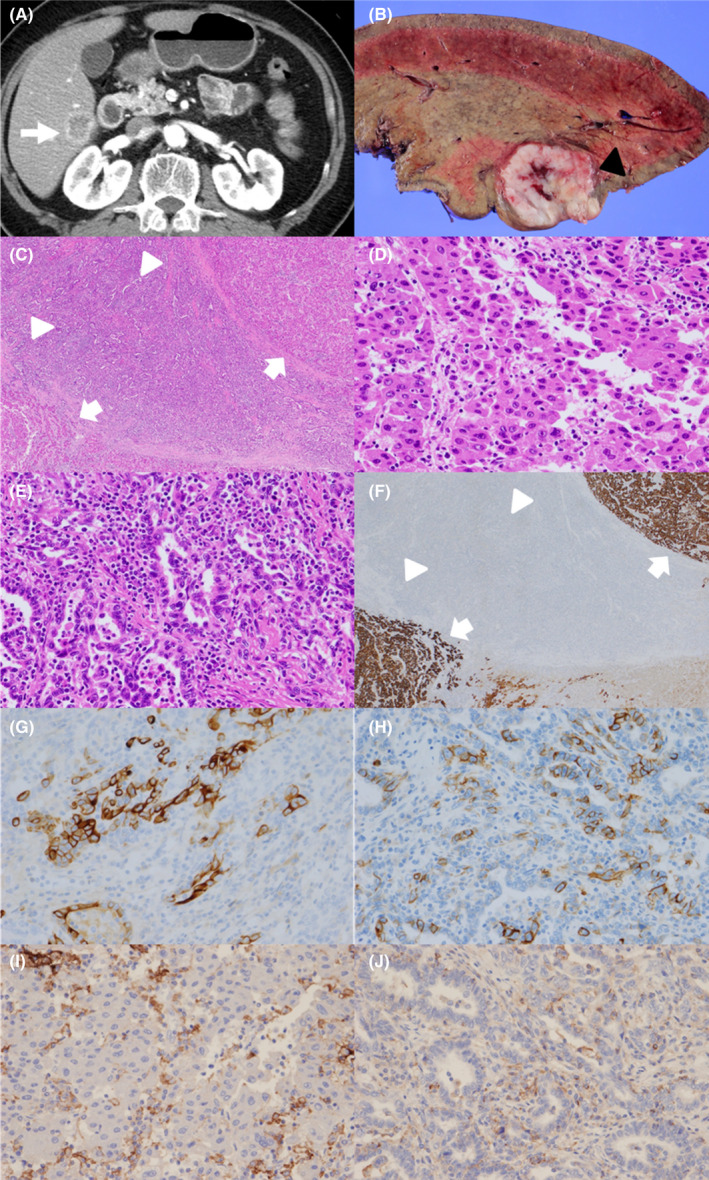
(A) Enhanced computed tomography performed before surgical treatment. The white arrowhead indicates the liver tumor. (B) A resected specimen. The black triangle indicates the liver tumor. (C) A low‐power‐field view of the histological examination revealed components of both hepatocellular carcinoma (HCC; white arrowheads) and cholangiocarcinoma (white triangles). (D) A high‐power‐field view of the HCC region. (E) A high‐power‐field view of the cholangiocarcinoma region. (F) Immunohistochemical staining showed that the HCC regions (white arrowheads) were positive and the cholangiocarcinoma regions (white triangles) negative for HepPar‐1. (G, H) The cholangiocarcinoma regions were positive for CK 19 and CD 56. (I) The HCC regions were negative for programmed death ligand‐1 (PD‐L1). (J) The cholangiocarcinoma regions were negative for PD‐L1

An 81‐year‐old man was referred to our hospital for the treatment of liver cancer. He had chronic liver disease due to significant alcoholic consumption. Enhanced computed tomography (CT) showed a liver tumor with marginal enhancement in the arterial phase at 28 mm in diameter in the right hepatic lobe (Figure 1A).

He underwent right hepatic lobectomy, and the tumor was completely resected. A gross examination showed a firm, solid yellowish white to tan‐gray mass (Figure 1B). A histological examination revealed components of both HCC and cholangiocarcinoma. Hematoxylin–eosin staining showed a trabecular‐sinusoidal growth pattern, morphologically consistent with HCC (Figure 1C,D), and glandular formations pattern, supportive of cholangiocarcinoma (Figure 1C,E). Immunohistochemical staining showed that the HCC regions were positive for HepPar‐1 (Figure 1F), and the cholangiocarcinoma regions were negative for HepPar‐1 (Figure 1F), positive for CK 19 (Figure 1G), and positive for CD 56 (Figure 1H). The patient was therefore pathologically diagnosed with CHC.

Both the HCC and cholangiocarcinoma regions were negative for PD‐L1 (Figure 1I,J). The microsatellite instability status was also negative. The surrounding liver tissue had steatosis with macrovesicular and fibrosis, suggesting alcoholic liver disease.

**FIGURE 2:**
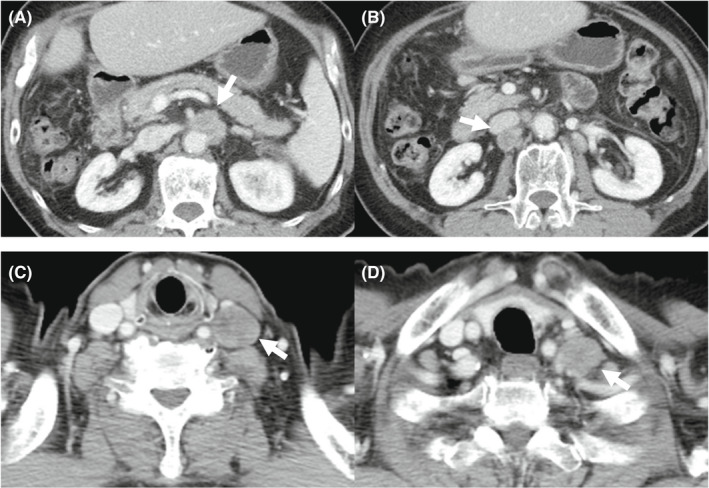
Enhanced computed tomography showed multiple lymph nodes metastases at 13 months after surgical treatment. (A) Lymph nodes around the abdominal aorta. (B) A lymph node behind the inferior vena cava. (C, D) Left supraclavicular lymph nodes

He underwent adjuvant chemotherapy with TS‐1 at 80 mg/day, but enhanced CT at 13 months after surgical treatment showed enlarged systemic lymph nodes around the abdominal aorta, behind the inferior vena cava, and at the anterior cervical region, suggestive of multiple lymph node metastases (Figure 2). He received systemic chemotherapy with gemcitabine and cisplatin (GEM/CDDP), but we had to discontinue the GEM/CDDP therapy due to a severe rash. Because lenvatinib was recommended for first‐line HCC treatment at that time, we administered lenvatinib at 8 mg/day for 4 months. However, we ultimately had to discontinue lenvatinib treatment due to grade 3 decreased appetite. Because Atez/Bev became available in Japan after the end of the lenvatinib treatment, we decided to administer combination therapy with Atez/Bev, following institutional review board approval and the acquisition of written informed consent from the patient.

**FIGURE 3:**
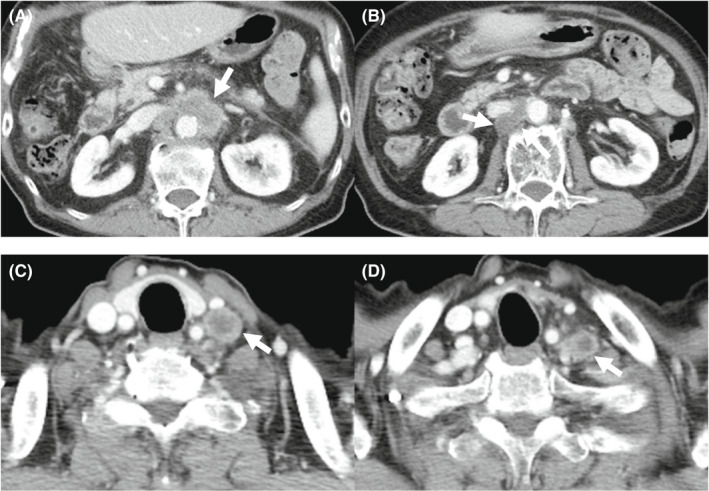
Enhanced computed tomography showed multiple lymph nodes metastases before Atez/Bev. The size of enlarged multiple lymph nodes remained almost the same and any new lesions were not detected. (A) Lymph nodes around the abdominal aorta. (B) A lymph node behind the inferior vena cava. (C, D) Left supraclavicular lymph nodes

**FIGURE 4:**
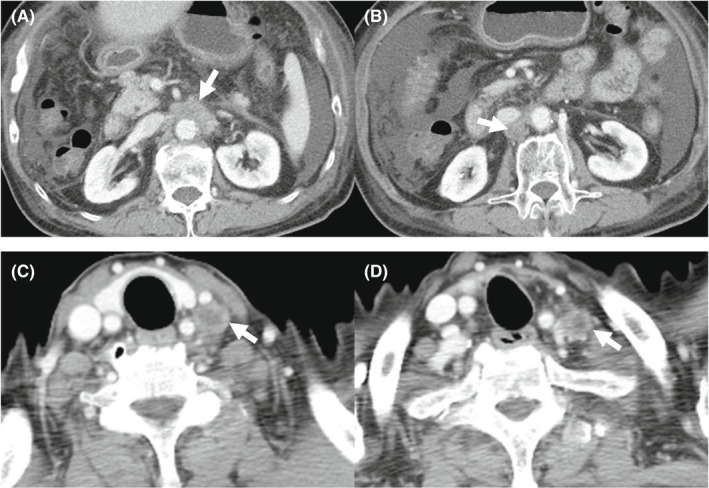
Enhanced computed tomography after two cycles of therapy showed mild shrinkage of enlarged lymph nodes, and the response rate per RECIST was determined to be stable disease. (A) Lymph nodes around the abdominal aorta. (B) A lymph node behind the inferior vena cava. (C, D) Left supraclavicular lymph nodes

**FIGURE 5:**
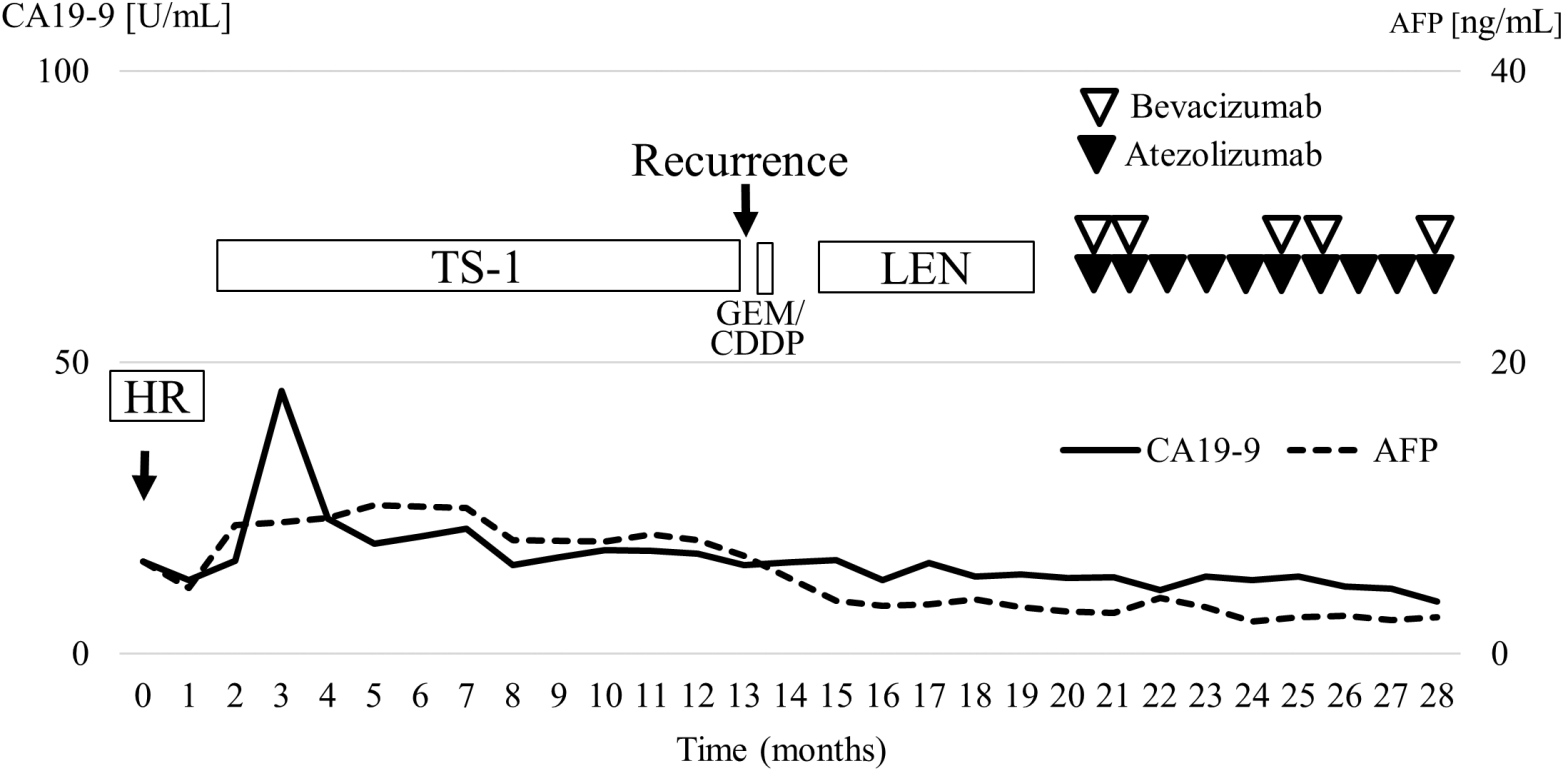
Clinical course. The solid line indicates CA19‐9, and the dotted line indicates AFP. Black and white triangles indicate the administration of atezolizumab and bevacizumab, respectively. Any tumor markers were not elevated during the clinical course. AFP, alfa‐fetoprotein; CA 19–9, carbohydrate antigen 19–9; HR, hepatic resection; GEM/CDDP, gemcitabine and cisplatin; LEN, lenvatinib

The laboratory findings before Atez/Bev treatment are shown in Table 1. The platelet count and hepatobiliary enzyme values were within the normal limits. Both hepatitis B surface antigen and hepatitis C virus antibody were negative. The serum levels of alpha‐fetoprotein (AFP), des‐gamma‐carboxy prothrombin (DCP), carcinoembryonic antigen (CEA), and carbohydrate antigen 19–9 (CA19‐9) were also within the normal ranges. Regarding the liver function, the Child‐Pugh classification was grade A (5 points), and the albumin‐bilirubin (ALBI) score was calculated to be −2.92, resulting in an assignment of ALBI grade 1. CT showed that the size of enlarged multiple lymph nodes remained almost the same and any new lesions were not detected (Figure 3). We started treatment at 1200 mg atezolizumab and 15 mg/kg of body weight bevacizumab every 3 weeks. CT after two cycles of therapy showed mild shrinkage of enlarged lymph nodes and the response rate per RECIST was determined to be stable disease (SD) (Figure 4). Due to the presence of ascites, we interrupted bevacizumab and continued atezolizumab monotherapy at the third, fourth, and fifth cycles. CT after five cycles showed a lasting tumor response. We resumed bevacizumab treatment combined with atezolizumab at the sixth and seventh cycles owing to the improvement of edema by diuretics. We deemed him to have stable diseases on CT after seven cycles of treatment. We continued atezolizumab monotherapy at the eighth and ninth cycles due to bevacizumab‐related adverse events, such as fatigue, decreased appetite, and proteinuria. After 10 cycles of treatment, CT showed the regrowth of enlarged lymph nodes and the response rate per RECIST was determined to the progressive disease (PD). Therefore, Atez/Bev treatment was discontinued, and the patient ultimately achieved a 7.5‐month progression‐free survival (PFS). Any tumor markers were not elevated during the clinical course. At 28 months after the initial hepatic treatment (15 months after initiation of systemic therapy), the patient was still alive. The overall clinical course is shown in Figure 5.

**TABLE 1:** Laboratory findings

Variables	Results	Variables	Results
Hb	12.6 g/dl	Ca	10.0 mg/dl
RBC	389 ×10^4^/μl	Mg	2.3 mg/dl
WBC	5850 /μl	CRP	0.02 mg/dl
Neut	4130 /μl	Glucose	91 mg/ml
PLT	16.6 ×10^4^/μl	HbA1c	5.6%
PT	91.2%	NH3	23 μg/dl
PT‐INR	1.05	TSH	2.794 μU/ml
APTT	35.4 s	FT3	2.69 pg/ml
D‐dimer	1.1 μg/ml	FT4	0.99 ng/dl
T.Bil	0.99 mg/dl	ACTH	45.2 pg/ml
AST	31 U/L	Cortisol	12.2 μg/ml
ALT	19 U/L	KL‐6	207 U/ml
LDH	184 U/L	CEA	2.8 ng/ml
ALP	266 U/L	CA19‐9	13.0 U/ml
γ‐GTP	46 U/L	AFP	2.9 ng/ml
ALB	4.13 g/dl	DCP	24 mAU/ml
BUN	14.6 mg/dl	HBs‐Ag	Negative
Cre	0.94 mg/dl	HCV‐Ab	Negative
Na	139 mmol/L	Urine TP/Cre	0.45
K	4.9 mmol/L	Child‐Pugh class A score 5	
Cl	104 mmol/L	ALBI grade 1 (score − 2.92)	

Abbreviations: ACTH, adrenocorticotropic hormone; AFP, alpha‐fetoprotein; ALB, albumin; ALBI, albumin‐bilirubin; ALP, alkaline phosphatase; ALT, alanine aminotransferase; APTT, activated partial thromboplastin time; AST, aspartate aminotransferase; BUN, blood urea nitrogen; Ca, calcium; CA19‐9, carbohydrate antigen 19–9; CEA, carcinoembryonic antigen; Cl, chlorine; Cre, creatinine; CRP, C‐reactive protein; DCP, des‐gamma‐carboxy prothrombin; FT3, free triiodothyronine; FT4, free thyroxine; γ‐GTP, gamma‐glutamyltransferase; Hb, hemoglobin; HbA1c, hemoglobin A 1c; HBs‐Ag, hepatitis B surface antigen; HCV‐Ab, hepatitis C virus antibody; K, potassium; LDH, lactate dehydrogenase; Mg, magnesium; Na, sodium; NH3, ammonia; PLT, platelet; PT, prothrombin time; PT‐INR, prothrombin time international normalized ratio; RBC, red blood cell; T.BIL, total bilirubin; TP, total protein; TSH, thyroid‐stimulating hormone; WBC, white blood cell.

## 3 DISCUSSION

We experienced a case of pathologically proven unresectable CHC treated with Atez/Bev treatment after hepatic resection. Given the rarity and heterogeneity of CHC, there is no established standard systemic chemotherapy for unresectable CHC patients. We administered adjuvant chemotherapy with TS‐1, but CT showed multiple lymph node metastases at 13 months after surgical treatment. We first administered GEM/CDDP, which is an established treatment for biliary tract malignant tumors, but we did not continue this therapy due to the development of a severe rash. Because no effective biliary tract regimen was available, we chose lenvatinib treatment, which has demonstrated the efficacy and safety for unresectable HCC and was recommended as the first‐line treatment at that time. Four months after the initiation of lenvatinib treatment, we discontinued lenvatinib therapy due to grade 3 decreased appetite. Because Atez/Bev became available in Japan after we had ceased lenvatinib treatment, we started Atez/Bev treatment and achieved a 7.5‐month PFS despite temporary bevacizumab interruption without severe adverse events. To the best of our knowledge, this is the first report of the administration of Atez/Bev to CHC patients.

According to a previous study evaluating 68 CHC patients receiving systemic therapies in a single institution,^10^ a gemcitabine plus platinum‐based regimen achieved an 8.0‐month PFS and 11.5‐month overall survival (OS), while gemcitabine plus fluorouracil and sorafenib regimens achieved a 6.6‐ and 4.8‐month PFS and 11.7‐ and 9.6‐month OS, respectively. A multicenter retrospective analysis reported from Japan showed that the median OS in patients receiving fluorouracil plus cisplatin, GEM/CDDP, and sorafenib were 11.9, 10.2, and 3.5 months, respectively.^11^ Sorafenib monotherapy was shown to be an unfavorable factor influencing the OS in a multivariate analysis.^11^ Another multicenter retrospective study conducted in French hospitals showed that the median PFS and OS were 9.0 and 16.2 months, respectively, in patients treated with gemcitabine plus platinum‐based chemotherapy.^12^ In the present case, Atez/Bev resulted in a 7.5‐month PFS, which was comparable with the therapeutic efficacy of the gemcitabine plus platinum‐based regimen. Therefore, Atez/Bev might be a viable treatment option for patients with unresectable CHC.

According to a randomized controlled trial (Imbrave150), Atez/Bev treatment resulted in a 6.8‐month median PFS in advanced HCC patients.^13^ In real‐world settings, the median PFS ranged from 5.0 to 6.5 months.^14^, ^15^, ^16^ Although both the HCC and cholangiocarcinoma regions were negative for PD‐L1 based on immunohistochemical staining, the median PFS in the present case was similar to that in Imbrave150 and these previous studies. Further analysis will be conducted to investigate the importance of the PD‐L1 expression in CHC patients. Another point of note is that we did not administer bevacizumab from three to five cycles and in eight and nine cycles due to bevacizumab‐related adverse events. Bevacizumab enhanced the efficacy of atezolizumab by supporting the maturation of dendritic cells, infiltration of T cells into tumors, and recognition of cancer cells.^17^ In addition, bevacizumab monotherapy has shown therapeutic efficacy for HCC in clinical settings.^18^, ^19^ Recently, we reported that early bevacizumab interruption was associated with a poor PFS and OS in patients receiving Atez/Bev.^20^ Accordingly, Atez/Bev may show good therapeutic efficacy for patients with CHC. A further study with a larger number of cases is warranted to confirm these points.

CHC is a rare form of primary liver cancer, and the reported percentage of CHC in primary liver cancer varies widely, from a rate of 1.0% according to a nationwide survey reported by Kudo et al.,^21^ to a rate of 3.6% reported by Jarnagin et al.,^22^ to a high of 14.2% in the case series study reported by Allen and Lisa.^23^ CHC is more common in men^4^ and individuals with cirrhosis or chronic liver disease, notably hepatitis B infection or significant alcoholic consumption,^8^, ^24^ than in others. These clinical features are similar to those of HCC and were compatible with the present case in terms of chronic liver disease due to significant alcohol consumption.

When CHC is diagnosed early, and the tumor extent is locally limited, surgical treatment may provide a long‐term survival. A population‐based study showed that the median survival in patients undergoing surgical treatment was 28 months, and prognostic factors were localized disease, tumor size <5 cm diameter, and surgical treatment.^4^ A single‐institution study reported that the PFS and OS in patients initially receiving surgical treatment was 10.4 and 25.7 months, respectively, whereas those in patients treated with hepatic directed therapy and systemic therapy were 8.3 and 16.0 months and 5.0 and 5.6 months, respectively.^5^ Another two reports conducted the single‐institution analysis of CHC patients receiving surgical treatment, indicating that the 5‐year OS rates were improved beyond 30%, although the 1‐year disease‐free survival ranged from 40% to 50%.^7^, ^8^ In the present case, 13 months have passed until the recurrence was observed after the initial surgical treatment, and the patient was still alive at 28 months after initial surgical treatment, which was presumed to be consistent with previous studies. Another point of note is that the tumor recurrence rate after surgical treatment was reported to be high. In the present case, we prescribed TS‐1 to prevent tumor recurrence after surgical treatment, but multiple lymph metastases were observed. Further studies to explore effective adjuvant chemotherapy should be conducted.

There are limited data on efficacy of liver‐directed therapies including transarterial chemoembolization, radiofrequency ablation, and microwave ablation.^25^ A retrospective study^5^ assessed 18 CHC patients treated with hepatic‐directed therapies, showing that the median PFS was 8.3 months and the median OS was 16.0 months. Another retrospective study^26^ evaluating 50 patients receiving hepatic‐directed therapies reported that the median survival was 12.3 months (95% CI 6.7–17.9 months) and 1‐, 2‐, 3‐, and 4‐years OS rates were 52%, 38%, 16%, and 12%, respectively. Although these studies were limited by small number of cases and retrospective manner, hepatic‐directed therapies might be effective for patients with unresectable CHC and recurrence following surgical resection.^25^

Several limitations associated with the present study warrant mention. First, this was a single case report; a large number of CHC patients will be needed to confirm the efficacy and safety of Atez/Bev treatment. Second, while Atez/Bev has demonstrated efficacy and safety for HCC patients, the efficacy of immune checkpoint inhibitors for biliary tract malignant tumors remains uncertain. We were unable to determine which of the two components (HCC or cholangiocarcinoma) was dominant based on the changes in tumor markers due to the lack of elevation of any tumor markers during the clinical course. Because recurrent lesions may not always have the same component as primary hepatic resected lesions, a pathological examination of the enlarged lymph nodes might be useful. One reason why we did not perform a needle biopsy is that the amount of obtained specimen was insufficient to evaluate the pathological features, including which of the two components was dominant. In addition, Atez/Bev is one of the most powerful HCC regimens and is recommended as the new‐first line treatment. Accordingly, treatment options may not markedly differ depending on the findings of the pathological examination at the metastasis site.

In conclusion, Atez/Bev exhibits therapeutic efficacy for CHC patients. Further investigations with large number of cases are warranted to investigate the efficacy and safety of Atez/Bev for unresectable CHC.

## AUTHOR CONTRIBUTIONS

All authors were involved in the preparation of this manuscript. NS examined patients, collected the data, and original draft preparation, TH examined patients, collected the data, reviewing, and editing, SN, YHazama, SY, YHachisu, YT, TY, NK, and YHosouchi examined patients and collected the data. KK involved in pathological analyses. HT and SK reviewed and edited the manuscript. TU involved in supervision. All authors read and approved the final version of the manuscript.

## CONFLICT OF INTEREST

Takeshi Hatanaka received lecture fee from Eisai. None of the other authors have potential conflicts of interest to declare.

## ETHICAL APPROVAL

This study was approved by the institutional review board of Gunma Saiseikai Maebashi Hospital (IRB No. 2021–010) and was conducted in accordance with the Declaration of Helsinki.

## CONSENT

Written informed consent was obtained from the patient to publish this report in accordance with the journal's patient consent policy.

## Data Availability

The data associated with the findings of this study are available from the corresponding author upon reasonable request.
